# Supplementary material for: Polyketide synthase gene clusters in the ericoid mycorrhizal fungus Oidiodendron maius: genome-wide prediction, expression profiling, and functional characterization of knockout mutants
Source: Front Plant Sci. 2026 Sep 16;17:1924430. doi: 10.3389/fpls.2026.1924430 (PMC13623903; doi:10.3389/fpls.2026.1924430)
Supplement: Supplementary file 1 [file SupplementaryFile1.zip › Supplementary Materials/Supplementary Folder 1.zip!/Oidma1_AssemblyScaffolds_Repeatmasked/knownclusterblast/cluster42/jgi.p_Oidma1_206020_mibig_hits.html]

| MIBiG Protein | Description | MIBiG Cluster | MiBiG Product | % ID | % Coverage | BLAST Score | E-value |
| --- | --- | --- | --- | --- | --- | --- | --- |
| BAE56596.1 |  | BGC0001123 | Aspirochlorine\_biosynthetic\_gene\_cluster | 54 | 99.0314769976 | 468 | 1e-131 |
| AAS92541.1 | SirI | BGC0001044 | Sirodesmin\_biosynthetic\_gene\_cluster | 37 | 98.5472154964 | 266 | 4.5e-71 |
| ctg1\_orf12 |  | BGC0000321 | Chaetocin\_biosynthetic\_gene\_cluster | 37 | 98.5472154964 | 262 | 1.1e-69 |
| CCE28988.1 | related\_to\_1-aminocyclopropane-1-carboxylate\_synthase | BGC0001365 | Clapurines\_biosynthetic\_gene\_cluster | 33 | 101.210653753 | 235 | 1.1e-61 |
| EAU36746.1 | conserved\_hypothetical\_protein | BGC0000292 | Acetylaranotin\_biosynthetic\_gene\_cluster | 41 | 61.0169491525 | 199 | 6.8e-51 |
